# Guillain-Barré syndrome outbreak in Tapachula temporally associated with the Zika virus introduction in Southern Mexico

**DOI:** 10.1017/S0950268822001625

**Published:** 2022-10-19

**Authors:** Ricardo Paul Rodríguez de la Rosa, J. Oggún Cano-Torres, Sonia Rosales, Anke Paula Kleinert, Arturo Gómez, Fernando George, José George, Mariana Piedad García, Gabriel Nájera-Cancino, Paola del Carmen Guerra-de-Blas, Pablo F Belaunzarán-Zamudio, John Beigel, Guillermo Miguel Ruiz-Palacios

**Affiliations:** 1 Hospital Regional de Alta Especialidad, Ciudad Salud, Tapachula, Chiapas, Mexico; 2 Departamento de Infectología, Instituto Nacional de Ciencias Médicas y Nutrición Salvador Zubirán, Mexico City, Mexico; 3 Clinton Health Access Initiative, Malaria Country Team, Panama City, Panama; 4 The Mexican Emerging Infectious Diseases Clinical Research Network (LaRed), Mexico City, Mexico; 5 Division of Microbiology and Infectious Diseases, National Institute of Allergy and Infectious Diseases (NIAID), Bethesda, MD, USA

**Keywords:** Clinical characteristics, Guillain-Barré syndrome, outbreak, zika infection

## Abstract

The Guillain-Barré syndrome (GBS) has been previously associated with Zika virus infection. We analysed the data from all the patients with GBS diagnosis that were admitted to a referral hospital, in Tapachula City during the period from January 2013 to August 2016, comparing the incidence of GBS according to the temporality of the Zika outbreak in Southern Mexico. Additionally, we described the clinical and epidemiological characteristics of the GBS patients admitted before or after the Zika outbreak. We observed a sharp increase in the number of patients hospitalised due to GBS from the time the first confirmed Zika cases appeared in Mexico. Clinically we observed GBS cases before zika outbreak had more frequently history of respiratory/gastrointestinal symptoms and GBS during zika outbreak had significantly more frequently recent history of rash/conjunctivitis. Although we cannot affirm that the increased cases of GBS have a specific aetiologic association with Zika, our results suggest that this observed outbreak of in Tapachula, might have been associated to the emerging Zika epidemic, locally and suggests that rare complications associated with acute infections (such as GBS) might be useful in the surveillance systems for emerging infections.

## Introduction

The Guillain-Barré syndrome (GBS) was first associated with Zika virus infection during the 2013–2014 outbreak in the French Polynesia [1]. Since then, GBS outbreaks have been reported in case-series and surveillance reports from Latin America following local Zika virus introduction and dissemination, corroborating the association between Zika virus and GBS [2]. Descriptions of clinical presentations, pathogenesis and epidemiological characteristics of Zika virus-associated GBS have shown heterogenous latency periods, and a broad variety of GBS-associated symptoms and severity [3]. Furthermore, a systematic review of the literature reported that as Zika virus disseminated northwards from South America, the incidence of GBS cases associated to Zika virus infection diminished to near zero in North America [4].

In Mexico the first confirmed Zika virus infection was reported in October 2015 in the southern region of the country, and by December of the same year 15 autochthonous and imported cases had been confirmed by real-time reverse transcription polymerase chain reaction (RT-PCR) [5]. At that time, the association of GBS with Zika virus infection was suspected but has not yet been confirmed. While no GBS cases were detected among PCR-confirmed ZIKV patients in the original description of the Zika introduction outbreak in Mexico [6], we aimed to describe the clinical and epidemiological characteristics of patients admitted to the Hospital Regional de Alta Especialidad – Ciudad Salud (Highly Specialized Regional Hospital or HRAE-CS) with GBS before the introduction of the Zika virus in Mexico (Jan, 2013 – Sep, 2015), and compare it with a cluster of GBS cases that presented as an outbreak starting in the weeks preceding the identification of the first Zika cases in Mexico (October 2015), and continued through this region during a Zika outbreak.

## Methods

The Hospital Regional de Alta Especialidad ‘Ciudad Salud’ (HRAE-CS) is a referral hospital located in the city of Tapachula, Chiapas in the southernmost region of Mexico. All patients with complex neurological diseases in the region of Tapachula are referred for care at this centre. Tapachula is a border city with Guatemala and the main point of entry for migrants from the Caribbean, Central, South America and Africa. We conducted an observational retrospective study, reviewing patients with GBS diagnosis admitted to the HRAE-CS between January 2013 and August 2016.

All diagnoses of GBS were made by neurologists using the Asbury criteria [7] and Brighton criteria, and the Mexican GBS Clinical Practice Guidelines [8, 9]. We retrieved socio-demographic and clinical information including age, sex, occupation, municipality, referral hospital, initial Hughes scale, Brighton level of certainty, recent history (up to 6 weeks) of infectious disease episodes prior to neurological symptoms onset and time elapse since infection and neurological deficit, GBS variant, clinical evolution and outcomes, CSF examination, electrodiagnostic studies, ICU date of admission and hospital length of stay from medical charts. Additionally, for patients requiring ventilatory support we collected the days since neurological deficit onset to intubation, days of mechanical ventilation and Hughes scale at discharge. Socio-demographic variables were collected from medical records. Historic cases (*n* = 24) were defined as those patients who were diagnosed between January 2013 through September 2015, while those temporarily associated to the Zika outbreak were diagnosed between October 2015 and August 2016. We estimated the incidence of GBS and describe its characteristics by period of diagnosis. We compared characteristics between groups using independent two-group comparisons of continuous variables with Mann-Whitney *U* test and categorical with the *χ*^2^ test. Statistical significance was set at *P* < 0.05. We obtained data on the cases of influenza, dengue, chikungunya, Zika, acute diarrhoeal and respiratory infections during the study period from the local office of the State Ministry of Health.

## Results

**Fig. 1.**
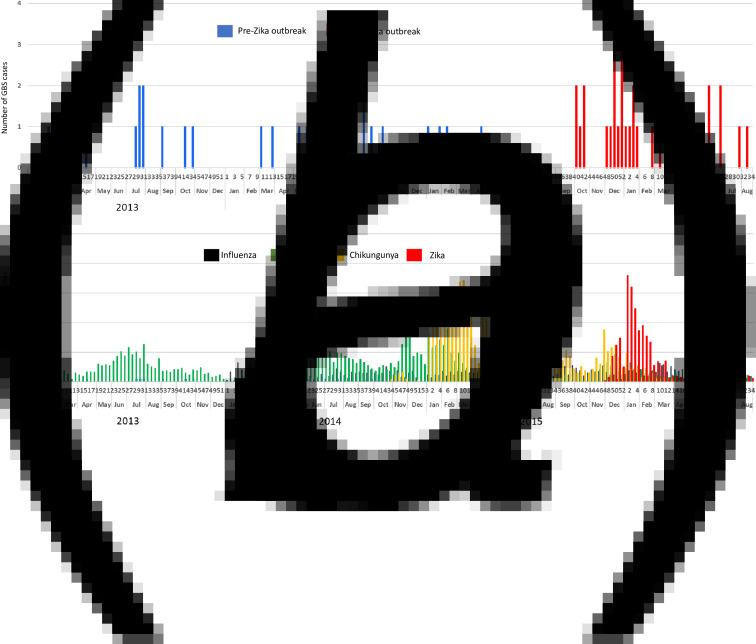
Epidemic curves of Guillain-Barré, Dengue, Chikungunya and Zika cases in Southern Mexico. (a) Patients with Guillain-Barré between January 2013 and August 2016; (b) Patients with Influenza, Dengue, Chikungunya and Zika between 2013 and 2016. *2013 Influenza Cases were not available.

We identified 54 cases of GBS admitted to HRAE-CS between January 2013 and August 2016. During the first 33 months (January 2013 to September 2015), twenty-four patients diagnosed with GBS (defined as historic cases) were admitted to HRAE-CS for an incidence of 2/100 000 persons: 11 in 2013, 9 on 2014 and the remaining 4 from January to September of 2015. In the following 10 months (October 2015 to August 2016), a substantial increase in the number of patients admitted to the HRAE-CS with GBS was observed (30 cases), for an incidence of 41/100 000 persons (Fig. 1). Half of these cases (*n* = 15) occurred between October and December 2015, and the first five cases were observed in October 2015. These GBS cases were distributed in two peaks: the first around the initial Zika outbreak and the second during the rainy season of 2016 (Fig. 1a). The initial GBS peak in October 2015 leads to the alert for the State and the National Ministries of Health of a potential Zika outbreak (Fig. 1a). During the study period, we observed no changes in reported frequency of dengue, influenza, acute diarrhoeal and respiratory infections (Fig. 1b and Supplementary Fig. S2). Chikungunya was initially reported the last weeks of 2014, with sustained transmission during 2015 and the largest peak during the summer and was gradually but steadily reduced during the last weeks of 2015 and first weeks of 2016.

The baseline clinical characteristics, type of GBS variant, diagnostic criteria, clinical evolution and outcomes did not differ between patients diagnosed before the Zika outbreak and after the Zika introduction to the region (see Supplementary Table S1). The only significant differences in clinical presentation between both periods was the frequency of an episode of rash and/or non-purulent conjunctivitis in the prior 6 weeks to the onset of neurological deficits: no patients diagnosed before October 2015 reported it, but 40% (*n* = 12) of patients developed rash and/or conjunctivitis prior to neurological symptoms onset (Supplementary Table S1). The geographical distribution of the cases was mostly along the state's coastline with the Pacific Ocean (Supplementary Fig. S1).

## Discussion

In this report, we describe a sharp increase in the number of patients admitted to the HRAE-CS due to Guillain-Barre Syndrome starting in October 2015. This surge was temporarily associated to the first confirmed Zika cases in Mexico, which were reported in this region. These findings contrast with the initial published description of the Zika outbreak at the national level [10].

Only 19 confirmed cases of GBS associated to Zika were reported and registered at the Mexican National Epidemiological Surveillance System during 2015 [10]. Similarly, in different nationwide studies published more recently and conducted between 2016 and June 2019, reported additional GBS cases with laboratory evidence of Zika infection [11–13]. Here, we describe an abrupt increase of cases of GBS from October 2015 to August 2016 in the region, that were temporally and geographically associated with the introduction of the Zika virus. Our data suggest that GBS associated to the introduction of the Zika epidemic were widely overlooked at the national level.

We also observed a change in the clinical pattern of the acute infections preceding these GBS episodes during the period of the initial Zika outbreak, as 40% of the outbreak-cases were clinically compatible with Zika infection (recent history of rash/conjunctivitis), and only 16.6% presented with respiratory/gastrointestinal symptoms. In contrast, the no rash/conjunctivitis cases were recorded for the historical cases, among whom a history of acute respiratory/gastrointestinal infection was more common. There were no other differences in clinical characteristics of outcomes between both periods. Patients with rash and conjunctivitis showed predominantly an acute ascending flaccid paralysis with a reduced time interval between rash/conjunctivitis and neurological symptoms onset. These results are consistent with a more recent, prospective, case-control study in Northeastern Mexico on which GBS cases during a Zika outbreak occurred primarily among those with recent history of confirmed, symptomatic ZikV infection, in comparison with those with asymptomatic confirmed Zika virus infection [13]. While we cannot establish a direct association with any specific, causal agent in this observational, epidemiological study, our results are consistent with previous published literature [11, 12], and indicates that the emergence of Zika in Mexico was epidemiologically associated with a temporal increase in GBS cases.

This report has several limitations, in part due to the retrospective nature of the study. While we observed that during the Zika period, recent history of rash/conjunctivitis was more frequently registered in the clinical charts of patients with GBS in comparison to previous years, clinicians might have been aware that Zika were soon to be circulating in the area or was already circulating, even before confirmed cases were reported, and biased the type of information they collected during clinical interviews. Furthermore, even if this is not a biased observation, we cannot affirm that the cases of GBS have a specific aetiologic association with Zika based on the presence of symptoms. In addition, we only included patients that were admitted to a single centre. However, this is a referral hospital, so most cases severe enough as to require hospitalisation were likely to be managed in this hospital. Finally, without having laboratory confirmation of possible aetiologic pathogens, we cannot firmly establish that this GBS outbreak was specifically associated with Zika virus infections in an area where other associated pathogens might co-circulate.

Despite these limitations, our study supports that the initial introduction of Zika to this region, contributed importantly to the GBS outbreak observed during 2015–2016. First, the abrupt increase of cases of GBS, was temporally and geographically associated with the introduction of the Zika virus, locally, over a stable background of dengue endemicity [14, 15]. Furthermore, dengue was limited to a low circulation in the Tapachula region from 2014 to 2016 (Fig. 1b). Second, while this is an endemic area for several arbovirosis, only Zika and dengue have been associated with GBS in Mexico, while GBS associated to chikungunya has only been exceptionally reported elsewhere [11–13, 16]. Soto-Hernández *et al*., studied 28 patients with GBS during an overlapping period (July–November 2016) in three other states (Guerrero, México State and Michoacán) which were tested by RT-PCR for ZIKV, dengue virus and chikungunya virus in serum, cerebrospinal fluid, urine and saliva. They found all RT-PCR tests for Dengue and chikungunya results negative [12]. We cannot rule out, of course, that an increase in the number of cases of other pathogens traditionally associated with GBS (e.g. Influenza, Campylobacter, etc) might have been occurring concomitantly, but we consider this scenario unlikely, and documented no substantial changes in the local frequency of influenza, acute diarrhoeal and respiratory infections during the study period (see figure Supplementary Fig. S2).

## Conclusion

In this tropical, Mexican region in the border with Guatemala, we observed a temporal association between GBS incidence and the Zika virus introduction. Our results suggest that the initial reports of the Zika virus outbreak in Mexico widely overlooked the occurrence of Zika associated GBS. Although we cannot affirm that the cases of GBS have a specific aetiologic association with Zika, the abrupt increase of cases of GBS temporally and geographically associated with the introduction of the Zika virus, suggests that this observed outbreak of GBS cases in Tapachula, might have been associated to the emerging Zika epidemic, locally. In this context, rare complications associated with acute infections (such as GBS) might be useful in the surveillance systems for emerging infections.

## Data Availability

The data presented in this study are available on request from the corresponding author. The data are not publicly available because a data-sharing agreement is needed to provide for: (1) a commitment to use the data only for research purposes and not to identify any individual participant; (2) a commitment to secure the data using appropriate computer technology; and (3) a commitment to destroying or returning the data after analyses are completed.

## Supplementary material

For supplementary material accompanying this paper visit https://doi.org/10.1017/S0950268822001625. click here to view supplementary material

## References

[1] Watrin L (2016) Guillain-Barré syndrome (42 cases) occurring during a Zika virus outbreak in French Polynesia. Medicine 95(14), e3257. Published online: April 8 2016. doi: 10.1097/MD.0000000000003257 27057874 PMC4998790

[2] Capasso A (2019) Incidence of Guillain-Barré syndrome (GBS) in Latin America and the Caribbean before and during the 2015–2016 Zika virus epidemic: a systematic review and meta-analysis. PLoS Neglected Tropical Diseases 13(8), e0007622. Published online: August 26 2019. doi: 10.1371/journal.pntd.0007622 31449532 PMC6730933

[3] Leonhard SE (2020) Guillain-Barré syndrome related to Zika virus infection: a systematic review and meta-analysis of the clinical and electrophysiological phenotype. PLoS Neglected Tropical Diseases 14(4), e0008264. Published online: April 27 2020. doi: 10.1371/journal.pntd.0008264 32339199 PMC7205322

[4] Del Carpio Orantes L. (2020) Guillain-Barré syndrome associated with Zika virus infection in the Americas: a bibliometric study. Neurologia (English Edition) 35(6), 426–429. doi: 10.1016/j.nrl.2018.05.001 30072272

[5] Díaz-Quiñonez JA (2016) Asian Genotype Zika virus detected in traveler returning to Mexico from Colombia, October 2015. Emerging Infectious Diseases 22(5), 937–939. doi: 10.3201/eid2205.160190 27088574 PMC4861540

[6] Guerbois M (2016) Outbreak of Zika virus infection, Chiapas State, Mexico, 2015, and first confirmed transmission by *Aedes aegypti* mosquitoes in the Americas. Journal of Infectious Diseases 214(9), 1349–1356. doi: 10.1093/infdis/jiw302 27436433 PMC5079363

[7] Asbury AK and Cornblath DR. (1990) Assessment of current diagnostic criteria for Guillain-Barré syndrome. Annals of Neurology 27(suppl.), S21–S24. doi: 10.1002/ana.410270707 2194422

[8] Sejvar JJ (2011) Guillain-Barré syndrome and Fisher syndrome: case definitions and guidelines for collection, analysis, and presentation of immunization safety data. Vaccine 29(3), 599–612. doi: 10.1016/j.vaccine.2010.06.003 20600491

[9] Social IMdS. Diagnóstico y Tratamiento Sindrome de Guillain- Barre Segundo y Tercer Nivel de Atención. Available at http://www.imss.gob.mx/sites/all/statics/guiasclinicas/089GRR.pdf (Accessed 8 March 2022).

[10] Salud Sd. Casos confirmados de Síndrome de Guillain-Barré asociado a Zika en México. Available at https://www.gob.mx/cms/uploads/attachment/file/390724/ZIKA_SxGB_060218.pdf (Accessed 8 March 2022).

[11] Grijalva I (2020) Zika and dengue but not chikungunya are associated with Guillain-Barré syndrome in Mexico: a case-control study. PLoS Neglected Tropical Diseases 14(12), e0008032. Published online December 17 2020. doi: 10.1371/journal.pntd.0008032 33332366 PMC7775118

[12] Soto-Hernández JL (2019) Guillain-Barré syndrome associated with Zika virus infection: a prospective case series from Mexico. Frontiers in Neurology 10, 435. 31114537 10.3389/fneur.2019.00435 PMC6502985

[13] Gongora-Rivera F (2020) Zika virus infection and Guillain-Barré syndrome in Northeastern Mexico: a case-control study. PLoS One 15, e0230132. 32214354 10.1371/journal.pone.0230132 PMC7098590

[14] Arredondo-García J (2020) Panorama epidemiológico de dengue en México 2000–2019. Revista Latinoamericana de Infectología Pediátrica 33, 78–83.

[15] Seceratría de Salud (2016) Simposio Binacional, Arbovirus y Fiebre Manchada en México. Available at https://www.epa.gov/sites/default/files/2016–11/documents/p2a.pdf (Accessed 6 September 2022).

[16] Lima MES (2019) Guillain-Barre syndrome and its correlation with dengue, Zika and chikungunya viruses infection based on a literature review of reported cases in Brazil. Acta Tropica 197, 105064. 31220435 10.1016/j.actatropica.2019.105064

