# Supplementary material for: Guillain-Barré syndrome outbreak in Tapachula temporally associated with the Zika virus introduction in Southern Mexico
Source: Epidemiol Infect. 2022 Oct 19;150:e181. doi: 10.1017/S0950268822001625 (PMC9980924; doi:10.1017/S0950268822001625)

**SUPPLEMENTARY MATERIAL**

**Supplementary Table 1.** **Demographics and medical history of participants**

|  |  | | |
| --- | --- | --- | --- |
|  | Patients enrolled pre-Zika outbreak (N=24) | Patients enrolled during Zika outbreak (N=30) | p-value |
| Age (years) |  |  |  |
| Mean (SD) | 41.70 (16.16) | 42.2 (15.57) | 0.898 |
| Sex |  |  |  |
| Female | 7 (29.17%) | 9 (30%) | 0.947 |
| Male | 17 (70.83%) | 21 (70%) |  |
| Civil Status |  |  |  |
| Single | 9 (37.50%) | 14 (46.67%) | 0.312 |
| Married | 13 (54.17%) | 16 (53.33%) |  |
| Divorced/Widow | 2 (8.33%) | 0 (0%) |  |
| Education Level |  |  |  |
| Did not go to school | 2 (8.33) | 2 (6.67) | 0.392 |
| Grades 1-6 (Primary) | 12 (50) | 10 (33.33) |  |
| Grades 7-9 | 4 (16.67) | 9 (30.00) |  |
| Grades 10-12 | 3 (12.50%) | 8 (26.67%) |  |
| University | 3 (12.50%) | 1 (3.33%) |  |
| Diabetes Mellitus 2 | 3 (!2.50%) | 3 (10.00) | 0.552 |
| High Blood Pressure | 6 (25.00%) | 5 (16.67%) | 0.510 |
| History of GI Infection | 7(29.17%) | 4 (13.33%) | 0.151 |
| Days between symptoms onset and neurologic symptoms | 4.6 (2.3) | 5.5 (1.91) | 0.572 |
| History of ARI | 5 (20.83) | 3 (10.00) | 0.265 |
| Days between symptoms onset and neurologic symptoms | 12.25 (6.64) | 13 (14.93) | 0.94 |
| History Rash/Conjunctivitis | 0 (0.00) | 12 (40.00) | 0.001 |
| Days between symptoms onset and rash | - | 7 (1-30) |  |
| **Diagnostic characteristics and outcomes** | | | |
| Initial Hughes Scale |  |  |  |
| 2 | 0 (0%) | 1 (3.33%) | 0.504 |
| 3 | 2 (8.33%) | 4 (13.33%) |  |
| 4 | 21 (87.50%) | 21 (70.0%) |  |
| 5 | 1(4.17%) | 4 (13.33%) |  |
| Brighton criteria (diagnostic certainty level) |  |  |  |
| 1 | 14(58.33%) | 8 (26.67%) | 0.043 |
| 2 | 4 (16.67%) | 3 (10.00%) |  |
| 3 | 5(20.83%) | 14 (46.67%) |  |
| 4 | 1(4.17%) | 5 (16.67%) |  |
| Flaccid paralysis (bilateral flaccid weakness) | 24 (100%) | 29 (96.67%) | 0.367 |
| GBS variant/type |  |  |  |
| Acute Flaccid Paralysis | 19 (79.17%) | 24 (80.00) | 1.00 |
| Pharyngeal-Cervico-Brachial/Miller Fisher | 5 (20.83%) | 6 (20.00) |  |
| Hyporeflexia/ areflexia | 24 (100%) | 30 (100%) |  |
| Cerebrospinal Fluid exam | 22 (91.67%) | 27 (90%) | 0.834 |
| Hyperproteinorraquia | 16 (76.19%) | 20 (80%) | 0.755 |
| Pleocytosis | 0 (0%) | 1 (4%) |  |
| Albuminocytological dissociation | 17 (80.95%) | 20 (83.33%) | 0.835 |
| Nerve Conduction Studies performed | 20 (83.33%) | 9 (30%) | <0.001 |
| Axonal | 12 (60%) | 5 (55.56%) |  |
| Demyelinating | 8 (40%) | 4 (44.44%) |  |
| Intravenous immune globulin treatment | 20 (83.33%) | 28 (93.33%) | 0.245 |
| Adverse Event to IVIG treatment | 1 (4.17%) | 0 (0.0%) | 0.259 |
| Length of hospitalization | 11.62 (14.08) | 15.26 (20.23) | 0.458 |
| UCI | 4 (16.67%) | 11 (36.67%) | 0.103 |
| Days in UCI | 10 (5.35) | 11 (6.6) | 0.191 |
| Ventilatory Support Initiation | 4 (16.67%) | 9 (30%) | 0.255 |
| Ventilatory Support Duration | 15.75 (9.21) | 32 (31.01) | 0.336 |
| Death | 2 (8.34) | 2 (6.67) | 0.816 |


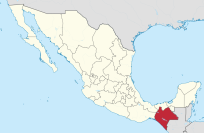
**Supplementary Figure 1. Municipalities of origin of patients with conjunctivitis / rash during the outbreak (October 2015-August 2016).**


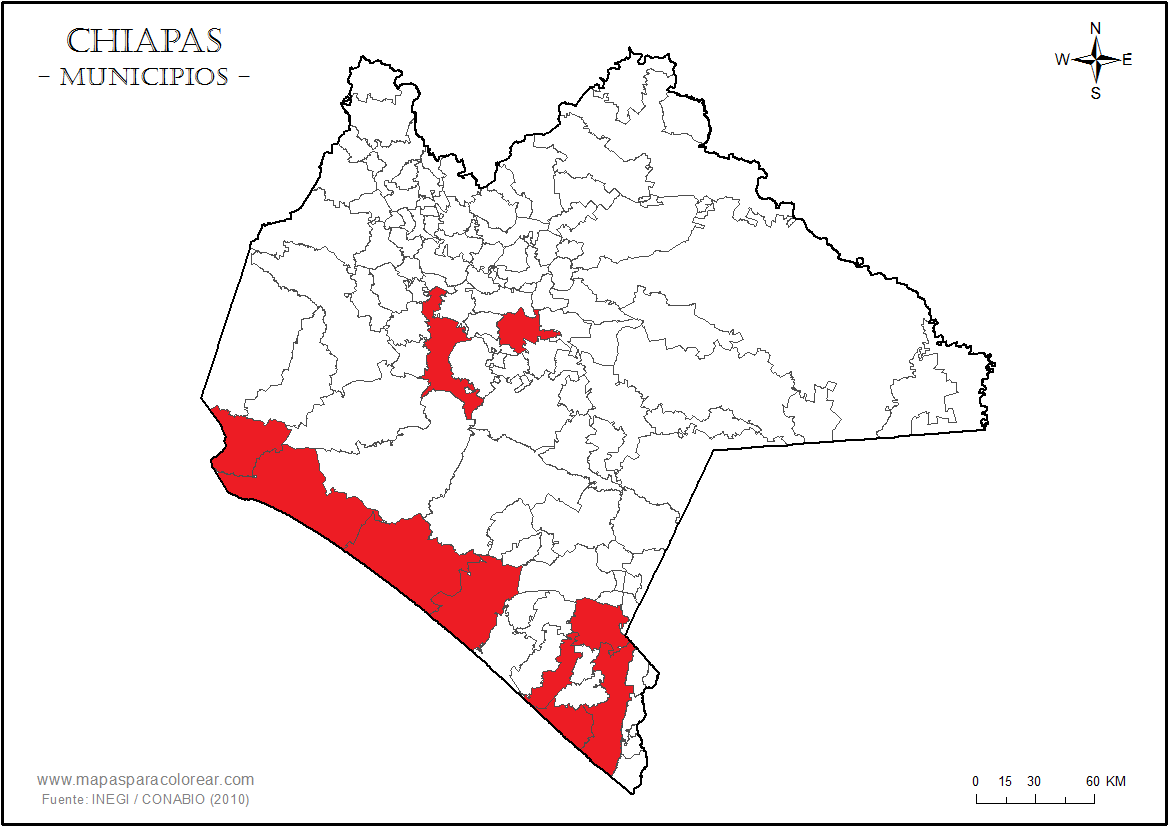


**Supplementary Figure 2. Frequency of acute diarrheal and respiratory infections in Southern Mexico (January 2013-August 2016).**


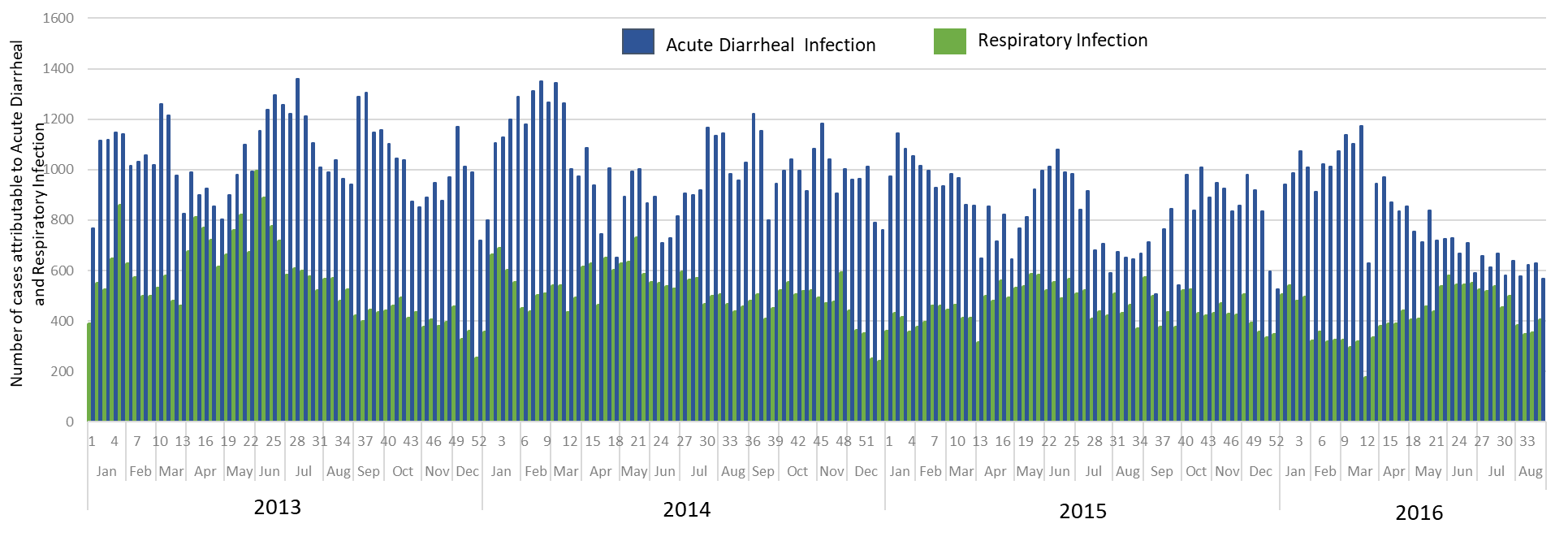

Supplement: Supplementary file 1 [file S0950268822001625sup001.docx]
